# Characterization of an Unknown Region Linked to the Glycoside Hydrolase Family 17 β-1,3-Glucanase of *Vibrio vulnificus* Reveals a Novel Glucan-Binding Domain

**DOI:** 10.3390/md20040250

**Published:** 2022-03-31

**Authors:** Yuya Kumagai, Hideki Kishimura, Weeranuch Lang, Takayoshi Tagami, Masayuki Okuyama, Atsuo Kimura

**Affiliations:** 1 Faculty of Fisheries Sciences, Hokkaido University, Hakodate 041-8611, Japan; i-dulse@fish.hokudai.ac.jp; 2 Research Faculty of Agriculture, Hokkaido University, Sapporo 060-8589, Japan; weranuch@abs.agr.hokudai.ac.jp (W.L.); tagami@abs.agr.hokudai.ac.jp (T.T.); okuyama@abs.agr.hokudai.ac.jp (M.O.)

**Keywords:** glucanase, *Vibrio*, carbohydrate-binding domain, glycoside hydrolase family 17

## Abstract

The glycoside hydrolase family 17 β-1,3-glucanase of *Vibrio vulnificus* (VvGH17) has two unknown regions in the N- and C-termini. Here, we characterized these domains by preparing mutant enzymes. VvGH17 demonstrated hydrolytic activity of β-(1→3)-glucan, mainly producing laminaribiose, but not of β-(1→3)/β-(1→4)-glucan. The C-terminal-truncated mutants (ΔC466 and ΔC441) showed decreased activity, approximately one-third of that of the WT, and ΔC415 lost almost all activity. An analysis using affinity gel containing laminarin or barley β-glucan revealed a shift in the mobility of the ΔC466, ΔC441, and ΔC415 mutants compared to the WT. Tryptophan residues showed a strong affinity for carbohydrates. Three of four point-mutations of the tryptophan in the C-terminus (W472A, W499A, and W542A) showed a reduction in binding ability to laminarin and barley β-glucan. The C-terminus was predicted to have a β-sandwich structure, and three tryptophan residues (Trp472, Trp499, and Trp542) constituted a putative substrate-binding cave. Linker and substrate-binding functions were assigned to the C-terminus. The N-terminal-truncated mutants also showed decreased activity. The WT formed a trimer, while the N-terminal truncations formed monomers, indicating that the N-terminus contributed to the multimeric form of VvGH17. The results of this study are useful for understanding the structure and the function of GH17 β-1,3-glucanases.

## 1. Introduction

Marine algae convert marine carbon into algal polysaccharides by photosynthesis. Algal polysaccharides are made up of a variety of glycans. The recycling of algal polysaccharides into carbon dioxide gives us a better understanding of the global marine carbon cycle [1]. Recently, the involvement of marine bacteria in this cycle has been gradually revealed [2]. Laminarin is a major glucose polymer found in marine environments [3]. Therefore, an understanding of the mechanisms underlying the degradation of large algal polysaccharides by enzymes and their modules is useful in order to produce sustainable and renewable raw materials for use in valuable compounds, feeds, and fuels [4].

Endo-β-1,3-glucanases catalyze the hydrolysis of internal β-(1→3)-glucosidic linkages. Endo-β-1,3-glucanases mainly belong to the enzyme families GH16, GH17, and GH3 [5]. The GH16 family is mainly composed of bacterial enzymes that catalyze β-(1→3)-glucan and β-(1→3)/β-(1→4)-glucan [6]. Laminarin is a natural β-(1→3)-glucan with occasional β-(1→6)-glucosyl branches found in marine micro- and macroalgae. Bacteria degrade and metabolize laminarin as a source of glucose [7,8,9,10,11,12]. The successive hydrolysis of laminarin by GH16 enzymes and GH3 enzymes, a family containing various kinds of glycosidases, has been reported [13,14,15,16,17,18]. On the other hand, the GH17 family is mainly composed of plant and fungal enzymes. They are classified as pathogen-related proteins [19,20,21] that contribute to the degradation and biosynthesis of the cell wall. Recently, many GH17 bacterial enzymes have been discovered due to the progress made in sequencing technology; however, the biological functions of bacterial enzymes are still unclear. Studies have shown that proteobacterial species produce an antibiotic biofilm via GH17 glucosyltransferase activity [22,23]. Another study reported that glucosyltransferase activity was modulated to a glucanase activity by a single mutation [24].

The CAZy database provides the taxonomic distribution of the GH17 family (cazy.org/IMG/krona/GH17_krona.html, accessed on 23 February 2022), revealing that a large number of bacterial enzymes are found within the phylum Proteobacteria (recently renamed Pseudomonadota). Within Proteobacteria, GH17 enzymes are commonly found within the genus *Pseudomonas*, with its diverse members and metabolism. While many *Vibrio* species, which belong to the class Gammaproteobacteria, have GH16 enzymes, a limited number possess enzymes of the GH17 family. The genome of *V. vulnificus* has been sequenced and annotated, and sequence analysis has revealed that one GH16 enzyme (VvGH16) and one GH17 enzyme (VvGH17) exist adjacently in the genome. One GH3 enzyme is located close to the other two enzymes. On the other hand, three GH16 (VbGH16A, VbGH16B, and VbGH16C) and one GH17 (VbGH17A) enzyme of *Vibrio breoganii* 1C10 have been characterized [25]. 

Several CAZymes have various domains in addition to the catalytic domain, including carbohydrate-binding modules (CBMs) [26,27,28]. These domains are involved in carbohydrate binding. Tryptophan is an important amino acid residue in carbohydrate binding [26]. The VvGH17 C-terminus has several tryptophan residues. Therefore, we predicted that this region may have functions, such as carbohydrate binding, that can increase the catalytic efficiency or specificity. In this study, we characterized GH17 β-1,3-glucanase of *V. vulnificus* to clarify the unknown region of the protein and found that the N- and C- terminal regions were affiliated with the assembly of monomeric subunits into the multimeric form and the affinity for the substrate, respectively.

## 2. Results

### 2.1. Bioinformatic Analysis of VvGH17

**Figure 1:**
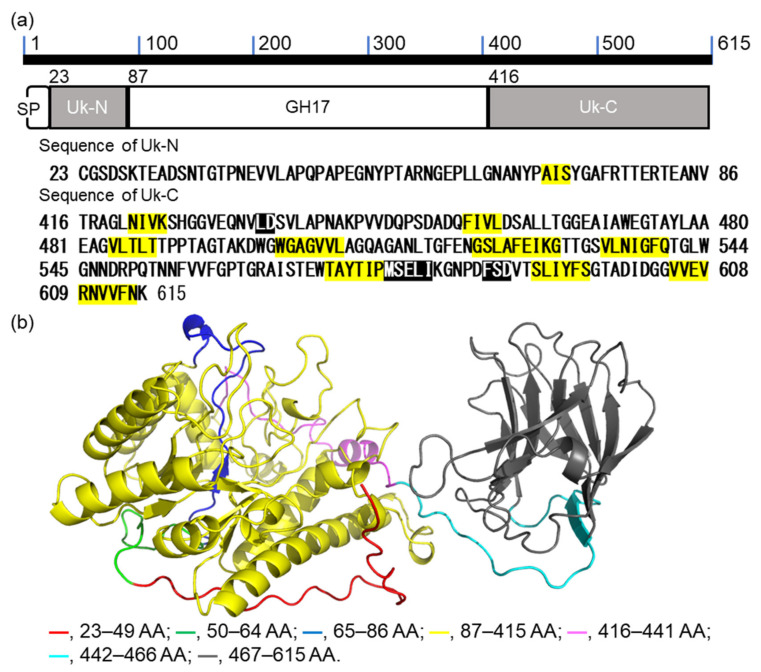
Sequence and predicted structure of VvGH17. (**a**) The scheme of VvGH17. SP—signal peptide; Uk-N—unknown N-terminal region; GH17—catalytic domain of GH17; Uk-C—unknown C-terminal region. Characters highlighted in yellow and those highlighted in black in the sequences are predicted α-fold and β-sheet structures, respectively. (**b**) Predicted three-dimensional structure. Colors are related to the truncated mutation region in this study.

VvGH17 is composed of 615 amino acids (AAs) comprising 1–22 AAs as signal peptides, 23–86 AAs as an unknown N-terminal region (Uk-N), 87–415 AAs as the GH17 domain, and 416–615 AAs as an unknown C-terminal region (Uk-C) (Figure 1a). Secondary structure prediction showed that Uk-N had a random coil structure, while Uk-C was composed of a β-sheet structure. The structure of VvGH17 was predicted using AlphaFold2 [29] (Figure 1b). The GH17 domain and C-terminus of Uk-C were predicted to have a (β/α)_8_ barrel structure and a β-sandwich structure, respectively. It was expected that the Uk-C structure possessed some function. Therefore, we attempted to characterize the impact of Uk-N and Uk-C on the catabolic properties of the enzymes.

### 2.2. Biochemical Properties of VvGH17

**Figure 2:**
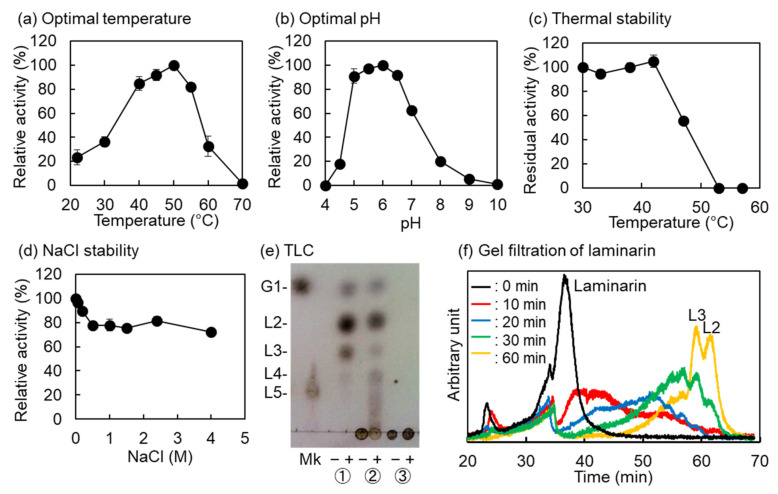
Characterization of VvGH17. (**a**) The effect of temperature on VvGH17 activity. The enzyme reaction was conducted in a mixture containing 50 mM MES buffer (pH 6.0), 1% (*w/v*) laminarin, and 0.02 mg/mL VvGH17 at 20–70 °C for 10 min. (**b**) The effect of pH on VvGH17 activity. The enzyme reaction was conducted in a mixture containing 1% (*w/v*) laminarin, 0.02 mg/mL VvGH17, and 100 mM Britton–Robinson buffer (pH 4.0–10.0) at 45 °C for 10 min. (**c**) The effect of temperature on VvGH17 stability. A mixture containing 50 mM MES buffer (pH 6.0) and 0.2 mg/mL VvGH17 was incubated at the indicated temperature for 30 min and placed on ice for 10 min. Then, the enzyme activity was assayed in a mixture containing 50 mM MES buffer (pH 6.0), 1% (*w/v*) laminarin, and 0.02 mg/mL VvGH17 at 45 °C for 10 min. (**d**) The effect of NaCl on VvGH17 activity. The enzyme reaction was conducted in a mixture containing 50 mM MES buffer (pH 6.0), 1% (*w/v*) laminarin, 0.02 mg/mL VvGH17, and 0–4.0 M NaCl at 45 °C for 10 min. (**e**) Thin layer chromatography (TLC) analyses of the hydrolysis products obtained using VvGH17. One microliter of each reaction mixture was applied for TLC analysis. Mk—marker of glucose and laminaripentaose; ‘−’—without VvGH17; ‘+’—with VvGH17. ①—curdlan; ②—laminarin; ③—β-glucan. (**f**) Gel filtration chromatography analysis for the hydrolysis of laminarin by VvGH17.

VvGH17 was produced in an *Escherichia coli* expression system and isolated using a TALON affinity resin (Takara Bio, Otsu, Japan). The purified enzyme (20 mg) was obtained from one liter of medium and showed a single band of approximately 63 kDa on SDS-PAGE. The optimal temperature and pH were 50 °C and 5.0–6.5, respectively (Figure 2a,b). The temperature required for the half inactivation of the hydrolysis activity of VvGH17 at 30 min was 47 °C (Figure 2c). The activity of VvGH17 decreased by approximately 80% in the presence of 0.5 M NaCl and retained the activity up to 4.0 M (Figure 2d). The specific activity of VvGH17 in optimal conditions was 65.5 U/mg. VvGH17 hydrolyzed curdlan (insoluble β-(1→3)-glucan) and laminarin, mainly producing laminaribiose, glucose, and laminaritriose; it did not hydrolyze barley β-glucan, which as β-(1→3)- and β-(1→4)-linkages (Figure 2e). The products of laminarin hydrolysis by VvGH17 were monitored from 0 to 60 min using gel filtration; the results revealed that VvGH17 hydrolyzed laminarin via an endolytic mechanism (Figure 2f). 

### 2.3. C-terminal-Truncated Mutant 

**Figure 3:**
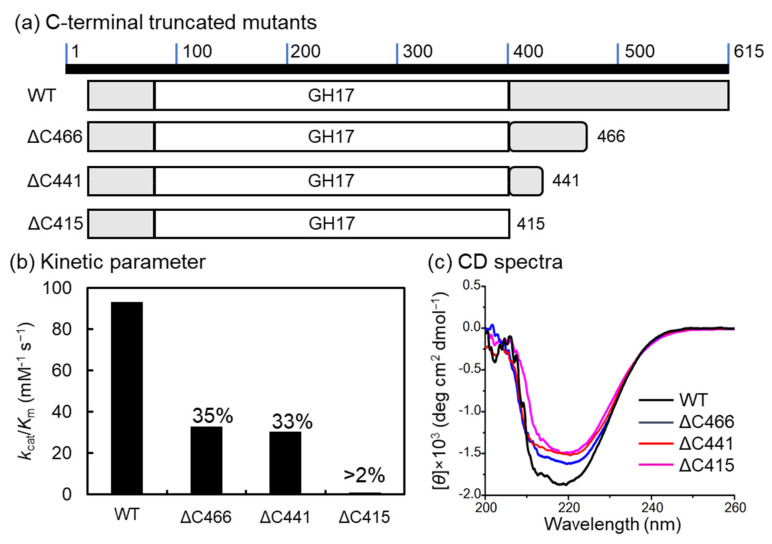
C-terminal-truncated mutants of VvGH17. (**a**) Scheme of C-terminal-truncated mutants. (**b**) Enzyme kinetics using laminarin as a substrate. The *k*_cat_/*K*_m_ value of the WT (93.0 mM^−1^ s^−1^) was set at 100%, and the relative values of the other mutants are indicated in the figure. (**c**) CD spectra of the WT and mutants. WT—black line; ΔC466—blue line; ΔC441—red line; ΔC415—pink line.

C-terminal-truncated mutants of VvGH17 were constructed for the characterization of Uk-C. The position from 87–415 AA was demonstrated as the conserved domain of GH17. Therefore, Uk-C was defined as the position from 416–615 AA in VvGH17, and the three C-terminal-truncated mutants (ΔC466, ΔC441, and ΔC415) were constructed (Figure 3a). Recombinant proteins of the three mutants were successfully expressed, and we evaluated the enzyme kinetics (Figure 3b, Table 1). The enzyme kinetics *k*_cat_/*K*_m_ of the WT toward laminarin was 93.0 mM^−1^ s^−1^, and 32.8 and 30.5 mM^−1^ s^−1^ for ΔC466 and ΔC441, respectively, which are approximately one-third of the *k*_cat_/*K*_m_ values in the WT. The *k*_cat_/*K*_m_ values of ΔC415 were less than 2% of those in the WT. To confirm whether the loss of activity in ΔC415 was derived from folding, the secondary structure was compared using circular dichroism (CD) spectroscopy (Figure 3c). The difference in the CD spectrum (deg cm^2^ dmol^−1^) between 210 and 230 nm may be a result of the deletion of the C-terminus in VvGH17. From these results, the truncation of the C-terminus in Uk-C (AA 442–615) resulted in a decrease in the catalytic efficiency of VvGH17, and the truncation of the whole Uk-C (416–615 AA) caused the loss of the majority of its activity, suggesting that this is an essential region.

### 2.4. Affinity Gel Analysis of C-Terminal-Truncated Mutants of VvGH17

**Figure 4:**
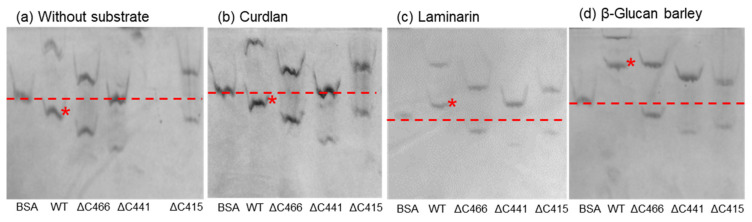
Affinity gel analysis of the WT and C-terminal-truncated mutants of VvGH17. (**a**) Affinity gel without substrate; (**b**) gel containing curdlan; (**c**) gel containing laminarin; (**d**) gel containing barley β-glucan. Asterisks show the WT monomer bands. The dashed lines show the mobility of BSA.

The truncation of the VvGH17 C-terminus revealed that this region affects the enzyme kinetics (*k*_cat_ and *K*_m_). This indicates that Uk-C has the potential for carbohydrate binding. To investigate the Uk-C function further, affinity gel analysis was performed (Figure 4). The WT and three C-terminal-truncated mutants showed two bands with and without substrates. The two bands were confirmed as monomers and oligomers (trimer) of the enzyme, as discussed in Section 2.7. Bovine serum albumin (BSA) was used as a marker protein for the mobility shift assay. No affinity toward curdlan was found in the tested enzymes, compared to the gel without substrate. The mobility of the WT monomer was clearly shifted from below the BSA band (without substrate) to upper the BSA band in the gels, confirming its affinity toward laminarin and β-glucan. 

### 2.5. Affinity Gel Analysis of Uk-C and Point Mutants of VvGH17

**Figure 5:**
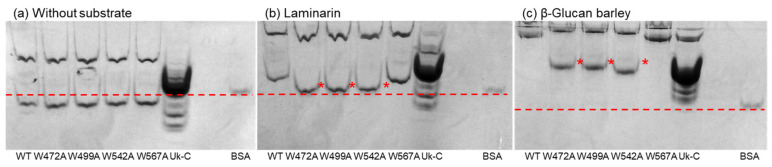
Affinity gel analysis of the WT, point mutants of VvGH17, and Uk-C. (**a**) Affinity gel without substrate; (**b**) gel containing laminarin; (**c**) gel containing barley β-glucan. Asterisks indicate the monomer bands showing different mobility. The dashed lines show the mobility of BSA.

The affinity of Uk-C and the C-terminus of VvGH17 toward laminarin and β-glucan was revealed. To confirm the important amino acids for substrate binding, point mutants of Uk-C and VvGH17 were constructed. Affinity toward substrates was also evaluated by the mobility as compared with BSA (Figure 5). The mobility of Uk-C in the gel containing laminarin and β-glucan was decreased compared to the gel without substrate. This indicated that Uk-C had a binding ability for laminarin and β-glucan. Tryptophan is an important amino acid for carbohydrate binding [26]. Therefore, we mutated four tryptophans in Uk-C to alanines (W472A, W499A, W542A, and W567A), and the affinity was evaluated by mobility shift assays. The WT and four mutants showed the same mobilities without a substrate. The mobilities of W472A, W499A, and W542A in the gel containing laminarin and β-glucan differed from those of the WT and W567A. The decreased mobility of W472A, W499A, and W542A indicated reduced binding ability, suggesting that the three tryptophans are essential amino acids for substrate binding.

### 2.6. Prediction of Uk-C Structure and Function

**Figure 6:**
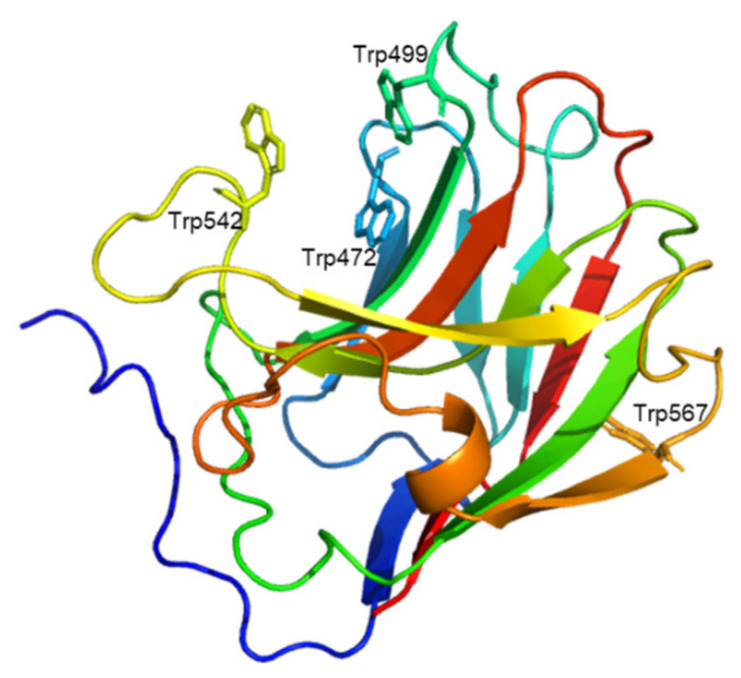
Structural prediction of the C-terminus of VvGH17. AA 441-615 of VvGH17 as predicted by AlphaFold2. Colors from blue to red show the sequence from AA 441 to 615. The locations of the four tryptophans (Trp472, Trp499, Trp542, and Trp567) are indicated.

We attempted to clarify the relationship between the predicted three-dimensional structure of Uk-C (AA 441–615 of VvGH17) and the binding ability of the mutants. The predicted Uk-C had two domains: the N-terminus of Uk-C (AA 416–462 of VvGH17) was predicted to be a linker between GH17 and the binding region, and the C-terminus of Uk-C (AA 463–615 of VvGH17) was predicted to be a β-sandwich structure with a possible carbohydrate-binding ability (Figure 1b). Three tryptophan residues (Trp472, Trp499, and Trp542) were located in the putative substrate binding region in the β-sandwich structure (Figure 6). On the other hand, the predicted structure suggested that Trp567 was located outside of the putative substrate binding region. The results of the mutation experiments agreed with the predicted structure. 

### 2.7. N-Terminal-Truncated Mutants of VvGH17

**Figure 7:**
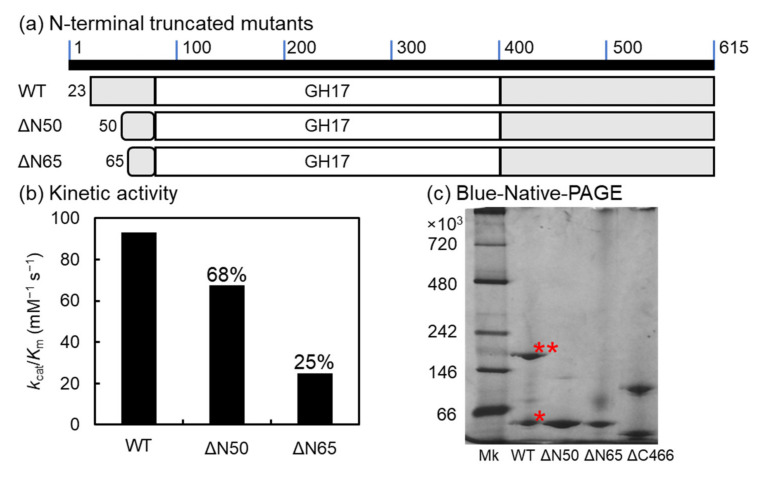
N-terminal-truncated mutants of VvGH17. (**a**) Scheme of N-terminal-truncated mutants. (**b**) Enzyme kinetics using laminarin as substrate. The *k*_cat_/*K*_m_ value of the WT (93.0 mM^−1^ s^−1^) was set at 100% and the relative values of the other mutants are indicated in the figure. (**c**) Blue native PAGE of the WT and N- and C-terminal-truncated mutants. *—WT monomer; **—putative WT trimer from molecular weight.

The catalytic domain of VvGH17 was mapped from AA 87 to 415, and AA 1–22 of VvGH17 were predicted as a signal peptide. The function of the N-terminus in VvGH17 (AA 23–86) was unclear. Therefore, we constructed two N-terminal-truncated mutants (ΔN50 and ΔN65) and evaluated their activity (Figure 7). The *k*_cat_/*K*_m_ values of ΔN50 and ΔN65 toward laminarin were 67.5 and 24.7 mM^−1^ s^−1^, respectively. The predicted three-dimensional structure of VvGH17 showed that the region of AA 51–65 was composed of the bottom of the (β/α)_8_ barrel structure (Figure 1b). Consequently, the loss of this region in the ΔN65 mutant led to structural instability, resulting in decreased catalytic efficiency (*K*_cat_/*k*_m_) (Table 1).

Two bands from the WT and C-terminal-truncated mutants were observed in native PAGE, as shown in Section 2.4. To confirm the assembly of monomeric subunits into the multimeric form, blue native PAGE was performed for the WT and N- and C-terminal-truncated mutants (Figure 7c). The WT and ΔC466 mutant clearly showed two bands (monomers and putative trimers from the molecular mass), while ΔN50 and ΔN65 showed a single band corresponding to the monomer. 

## 3. Discussion

In this study, we characterized the GH17 enzymes of *V. vulnificus* using an *E. coli* expression system. Functionally, GH17 enzymes have been reported to be β-(1→3)-glucan hydrolases and transglycosylases. In particular, endo-type β-(1→3)-glucanases are classified into the enzyme commission (EC) number EC 3.2.1.6 endo-1,3(4)-β-glucanase; EC 3.2.1.39 represents glucan endo-1,3-β-D-glucosidase; and EC 3.2.1.73 indicates licheninase. Our results showed that VvGH17 was classified into EC 3.2.1.39 due to its endolytic mechanism and specificity for β-(1→3)-glucan.

VvGH17 produced laminaribiose as the main product regardless of soluble and insoluble β-(1→3)-glucans, showing its potential for oligosaccharide production. The amino acid identity between VvGH17 and VbGH17A from *V. breoganii* 1C10 was low (42% identity). VbGH17A contains a signal peptide, the GH17 catalytic domain, and an unknown region of the C-terminus from amino acid (AA) 411 to 634 [18]. The catalytic domains of VvGH17 and VbGH17A shared 56% identity; however, the identity between the C-termini was low (21%). The hydrolysis products of VbGH17A were oligosaccharides, which were larger than a degree of polymerization (DP) of 4. *V. breoganii* 1C10 has four endo-type β-(1→3)-glucanases, which presumably show synergic activity during the hydrolysis of β-(1→3)-glucans. On the other hand, *V. vulnificus* has two endo-type β-(1→3)-glucanases. We expected several enzymes; however, there was only GH16, which had 57% identity with VbGH16A and produced mainly DP 3 and 4. The GH3 enzymes of *Vibrio* sp. have been shown to have activity toward laminaribiose [30]. Therefore, *V. vulnificus* may metabolize β-(1→3)-glucan by producing small DP oligosaccharides using two endo-type enzymes and then hydrolyzing them using the GH3 enzyme.

**Figure 8:**
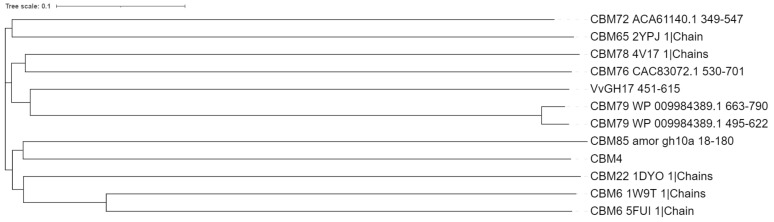
Phylogenetic tree of VvGH17 C-terminal domain and relatives. The tree was constructed based on ClustalW pairwise sequence alignment using the iTOL visualizing software. The following amino acid sequences were used: CBM4, laminarinase 16A from *Thermotoga maritima*, accession number (AN)—AAD35118, protein data bank (PDB) —1GUI [35]; CBM6, β-1,3-glucanase from *Alkalihalobacillus halodurans* C-125, AN—BAB03955, PDB—1W9T [36]; CBM6, endo-β-1,3-glucanase from *Zobellia galactanivorans*, AN—CAZ95067, PDB—5FUI [36]; CBM22, xylanase Xyn10B from *Acetivibrio thermocellus* YS, AN—CAA58242, PDB—1DYO [37]; CBM65, endoglucanase (EcCel5A) from *Eubacterium cellulosolvens* 5, AN—BAE46390, PDB—2YPJ [38]; CBM72, endoglucanase from uncultured microorganism, AN—EU449484 [39]; CBM76, GH44 from *Ruminococcus flavefaciens*, AN—AAA95959 [34]; CBM78, GH5 from *R. flavefaciens*, AN—WP_009983134, PDB—4V17 [34]; CBM79, GH9 from *R. flavefaciens*, AN—WP_009984389 [34]; CBM85, GH10 xylanase from metagenomic data, AN—MH727997 [27]; and VvGH17 from 451–615 AA. His-tag sequences from CBM6_5FUI, CBM65_2YPJ, and CBM78_4V17 were removed. The alignment of the tree and VvGH17 and CBM79 are shown in Figure S1 and Figure S2, respectively.

In this study, we identified the Uk-C as a carbohydrate binding-domain. VvGH17 formed a trimer, and the complex structure also showed carbohydrate binding activity. It can be concluded that the N-terminal region is affiliated with the trimerization of VvGH17. A more detailed structure-function analysis is needed. Uk-C showed binding activity with β-(1→3)-glucan and β-(1→3)/β-(1→4)-glucan and no binding activity with curdlan, an insoluble triple helix β-(1→3)-glucan. The TLC results indicated that VvGH17 hydrolyzed curdlan as well as laminarin without the assistance of Uk-C (Figure 2e). This study investigated the affinity of Uk-C for insoluble curdlan, but not the affinity for curdlan gel. Therefore, the binding specificity of Uk-C for a linear β-(1→3)-glucan should be further investigated. Curdlan is produced by the soil bacterium *Agrobacterium* sp., and *V. vulnificus* is a marine bacterium. Therefore, Uk-C might be specific for the marine polysaccharide laminarin. Laminarin is a soluble β-(1→3)-glucan with a β-(1→6)-glycosyl side chain. The difference in polysaccharide structure could affect the binding specificity. CBMs are classified into three types by their ligand binding sites. A-type CBMs recognize crystalline polysaccharide-like cellulose and chitin. B-type CBMs recognize a single glycan by binding a cleft or groove. C-type CBMs recognize the glycan terminus by binding pockets [26]. The Uk-C structure was predicted to be a B-type groove with three tryptophan residues. We confirmed that the mutations constituted the groove. Therefore, the mutation of tryptophan residues decreased the glucan binding ability. A BLAST search of Uk-C (416–615 AA) showed identity with CBM domains linked to other GH17 enzymes. A high AA identity of more than 95% was shared with the CBM domains of the GH17 enzymes from *Vibrio* sp., including *V. fluvialis*, *V. cholerae*, *V. metoecus,* and *V. metschnikovii*. A total of 30–70% identities were shared with enzymes from bacterial species, such as *Enterovibrio* sp., *Porticoccaceae* sp., *Bacteroidetes* sp., and *Grimontia* sp. (Table S1). The CBMs were classified into 89 families in the CAZy database (accessed on 16 February 2022). Among them, an affinity for β-(1→3)-glucan or β-(1→3)/β-(1→4)-glucan was demonstrated by 18 families: CBM4, 6, 11, 22, 28, 39, 43, 52, 54, 56, 65, 72, 76, 78, 79, 80, 81, and 85. CBM43, linked to eukaryotic GH17 enzymes, generally consists of 90-100 AAs [31]. The C-terminus of VvGH17 consisted of 150 AAs. Therefore, the sequences of CBMs belonging to nine families with around 150 AA residues were selected and aligned using ClustalW (https://www.genome.jp/tools-bin/clustalw, accessed on 23 February 2022) after removing the His-tag sequence, and we visualized the tree using iTOL [32] (Figure 8). The C-terminus of VvGH17 showed the closest relationship to the CBM79 cluster. The AA identity between Uk-C and CBM79 was 15.6-17.2% (Figure S1, Supplementary Materials). The complete genome sequence of *V. vulnificus* was determined in 2011 [33], and CBM79 family was recorded in 2016 [34]; however, Uk-C has not been included. In addition, the Uk-C sequence was not hit by a BLASTP search using CBM79 as query sequence. The distance of VvGH17 and CBM79 was similar to other CBM clusters (Figure 8), indicating that Uk-C is a novel soluble β-(1→3)-glucan and β-(1→3)/β-(1→4)-glucan-binding protein.

## 4. Materials and Methods

### 4.1. Materials

Curdlan was purchased from Fujifilm Wako Pure Chemicals Industries Ltd. (Osaka, Japan); laminarin (*Laminaria digitata*) was from Sigma-Aldrich Corp. (St. Louis, MO, USA); and β-glucan (barley; medium viscosity) was from Megazyme International Ireland Ltd. (Bray, Ireland). Laminaripentaose was prepared by the hydrolysis of curdlan with KfGH64 [40]. All the other reagents were purchased from Wako Pure Chemical Industries (Osaka, Japan).

### 4.2. Bioinformatic Analysis of VvGH17

The GH17 gene from *Vibrio vulnificus* (hypothetical protein AOT11_01225) was obtained from GenBank (accession no. ASM98089.1). The putative conserved domain was searched using BLASTP [41]. The signal peptide was predicted using the SignalP 4.1 server [42]. Secondary structure prediction was performed using the PSIPRED server [43] and the structure was predicted by AlphaFold2 [29]. Homolog proteins of the C-terminus of VvGH17 (416–615 AA) were searched by BLASTP with the standard algorithm, excluding *Vibrio vulnificus* (taxid: 672); we also removed query covers of less than 40%. A phylogenetic tree was constructed by pairwise sequence alignment.

### 4.3. Construction, Expression, and Purification of VvGH17

The expression plasmid of the gene putatively encoding β-(1→3)-glucanase (VvGH17) was constructed as follows: a codon-optimized mature *Vvgh17* gene was synthesized (Eurofins Genomics) for expression in *E. coli* harboring *Nde*I and *Hin*dIII sites at 5′ and 3′, respectively. Then, the *Vvgh17* gene was cloned into the *Nde*I-*Hin*dIII site of pET28a to construct an expression vector of pET28a(VvGH17). The recombinant protein was produced in *E. coli* BL21-RIL (DE3) cells (Agilent Technologies, Palo Alto, CA, USA) harboring pET28a(VvGH17) and was purified as previously described [44]. The protein concentrations were determined by absorbance at 280 nm using the molar extinction coefficients for VvGH17 [45].

### 4.4. Construction of VvGH17 Mutants

The C-terminal-truncated mutants (ΔC466, ΔC441, and ΔC415), N-terminal-truncated mutants (ΔN50, ΔN65, and UK-C), and point mutants (W472A, W499A, W542A, and W567A) were constructed by polymerase chain reaction using PrimeSTAR MAX DNA polymerase (Takara Bio, Otsu, Japan), primers (Table 2), and pET28a(VvGH17) as a template. 

### 4.5. VvGH17 Standard Activity Assay

VvGH17 activity was determined at 45 °C for 10 min with an appropriate amount of enzyme, 1% (*w/v*) laminarin, and 50 mM 2-morpholinoethanesulfonic acid (MES; pH 6.0). The amount of reducing sugars was determined using the dinitrosalicylic acid method (DNS) [46]. One unit of activity was defined as the amount of enzyme that liberated reducing sugars equivalent to 1.0 μmol glucose per minute. The optimal temperature of VvGH17 was measured as follows: a reaction mixture containing 1% (*w/v*) laminarin and 50 mM MES (pH 6.0) was incubated at 22–70 °C for 10 min. The optimal pH of VvGH17 was measured as follows: a reaction mixture containing 1% (*w/v*) laminarin and 100 mM Britton–Robinson buffer (a mixture containing sodium acetate buffer, sodium phosphate buffer, and glycine–NaOH buffer; pH 4.0–10.0) was incubated at 45 °C for 10 min. Temperature stability was determined by measuring the residual activity after incubation in 50 mM MES (pH 6.0) at 30–57 °C for 30 min. The effect of NaCl was determined using a mixture containing 1% (*w/v*) laminarin, 50 mM MES (pH 6.0), and 0–4.0 M NaCl at 45 °C for 10 min. The *V*_max_ and *K*_m_ with laminarin (0.5–40 mg/mL) were determined by the standard Michaelis–Menten equation using nonlinear regression (Origin Software, Lightstone Corp., Tokyo, Japan). All the activity assays were performed in triplicate.

### 4.6. Analysis of Hydrolysis Products by TLC and Gel Filtration Chromatography

The products of VvGH17 hydrolysis were analyzed by TLC using a silica gel 60 plate (Merck). The substrates (curdlan, laminarin, and β-glucan: 10 mg/mL) were hydrolyzed with 0.01 U/mL of VvGH17 for 24 h, and the reaction was terminated by heating at 100 °C for 10 min. The hydrolysis products (1 μL) were developed in ethyl acetate, acetic acid, and water (2:2:1, *v/v/v*); sugars were detected by spraying a solution of 10% (*v/v*) sulfuric acid in ethanol and then heating at 100 °C for 10 min.

The distribution of the hydrolysis products of laminarin was analyzed using high-performance liquid chromatography (HPLC) with a Superdex Peptide 10/300 GL column (GE Healthcare UK Ltd., Little Chalfont, UK) and a Corona Charged Aerosol Detector (Thermo Scientific Inc., Chelmsford, MA, USA). Laminarin (10 mg/mL) was hydrolyzed with 0.01 U/mL of VvGH17 from 0 to 60 min, and the reaction was terminated by heating at 100 °C for 10 min. The samples were eluted using water with a flow rate of 0.3 mL/min.

### 4.7. CD Spectroscopy

The secondary structures of VvGH17 were determined by CD spectroscopy using a J-720WI spectrometer (Jasco Corp. Tokyo, Japan). The proteins were dissolved at a final concentration of 0.1 mg/mL in 50 mM MES buffer (pH 6.0). The spectra were acquired at 37 °C using a 0.2 cm cuvette. The molar ellipticities (per residue) were calculated using the equation [*θ*] = 100(*θ*)/(*lcN*), where [*θ*] is the molar ellipticity per residue, (*θ*) is the observed ellipticity in degrees, *l* is the optical path length in centimeters, *c* is the molar concentration of the protein, and *N* is the number of residues in the protein.

### 4.8. Polyacrylamide Gel Electrophoresis (PAGE) Analysis

The assays for the binding activity of the proteins were performed by affinity gel electrophoresis, according to the procedure described by Zhang et al. [47]. A stacking gel containing 3 wt% polyacrylamide in 1.5 M Tris-HCl buffer (pH 8.3), a native gel with 12 wt% polyacrylamide containing 0.1 wt% polysaccharides (curdlan, laminarin, and barley β-glucan), and a control gel without polysaccharides were prepared. Each protein (1 μg) was loaded onto the gel, and the gels were electrophoresed at 4 °C and 100 V for 3 h. The gels were then stained with Coomassie brilliant blue G-250 for protein visualization.

Blue native PAGE was performed using a 5–10% gradient gel at 4 °C and 150 V held constant for 3.5 h using an anode buffer (50 mM tricine, 15 mM bis-Tris/HCl, pH 7) and cathode buffer (50 mM tricine, 15 mM bis-Tris/HCl, pH 7, 0.02% (*w/v*) Coomassie blue G250).

## 5. Conclusions

In this study, we characterized the unknown domains of the GH17 β-(1→3)-glucanase of *V. vulnificus*. The WT formed a trimer, but the N-terminal truncations formed monomers. Therefore, the N-terminus contributes to the assembly of monomeric subunits into the multimeric form of VvGH17. The C-terminal region showed an affinity for β-(1→3)-glucan and β-(1→3)/β-(1→4)-glucan. The C-terminus was predicted to have a β-sandwich structure, and three tryptophan residues (Trp472, Trp499, and Trp542) were located at the substrate binding site using mutational analysis. A BLAST search revealed that the C-terminal region of GH17 was conserved among Gammaproteobacteria. The results of this study are useful for understanding bacterial GH17 enzymes and oligosaccharide preparation.

## Figures and Tables

**Table 1:** Enzyme kinetics of VvGH17 and mutants using laminarin as a substrate.

	*k*_cat_/*K*_m_ (mM^−1^ s^−1^)	*k*_cat_ (s^−1^)	*K*_m_ (mM^−1^)
VvGH17	93.0	148	1.60
ΔC466	35.3	81.7	2.49
ΔC441	32.7	87.6	2.87
ΔC415	0.2	1.4	9.39
ΔN50	67.5	143.3	2.12
ΔN65	24.7	86.8	3.52

**Table 2:** Sequences of the primers used in this study.

Primer Name	Primer Sequence (5′-3′)	Purpose
ΔC466-S	TGATGGCAAGCTTGCGGCCGCACTC	Truncation of 149 AAs from C-terminus
ΔC466-AS	GCAAGCTTGCCATCAAACGCGCCGGC	
ΔC441-S	GACCGGCAAGCTTGCGGCCGCACTC	Truncation of 174 AAs from C-terminus
ΔC441-AS	GCAAGCTTGCCGGTCAGTAATGCACT	
ΔC415-S	TGCTCCGTGAAAGCTTGCGGCCGCACTC	Truncation of 200 AAs from C-terminus
ΔC415-AS	GCAAGCTTTCACGGAGCAAGAACGGAAT	
ΔN50-S	CCATATGGGCAACTATCCGACAGCT	Truncation of 50 AAsf rom N-terminus
ΔN50-AS	TAGTTGCCCATATGGCTGCCGCGCGG	
ΔN65-S	CCATATGGGCAACGCGAATTATCCG	Truncation of 65 AAs from N-terminus
ΔN65-AS	GCGTTGCCCATATGGCTGCCGCGCGG	
UK-C-S	CCATATGGCCGGCGCGTTTGATGGC	Truncation of 410 AAs from N-terminus
UK-C-AS	GCGCCGGCCATATGGCTGCCGCGCGG	
W472A-S	ATCGCAgcgGAAGGTACCGCCTATCTG	Mutation of Trp472 to Ala472
W472A-AS	ACCTTCcgcTGCGATCGCTTCCCCGCC	
W499A-S	TGGGGTgcgGGAGCGGGCGTCGTGCTC	Mutation of Trp499 to Ala499
W499A-AS	CGCTCCcgcACCCCAGTCTTTTGCAGT	
W542A-S	GGCCTGgcgGGCAACAACGACCGTCCG	Mutation of Trp542 to Ala542
W542A-AS	GTTGCCcgcCAGGCCGGTCTGAAATCC	
W567A-S	ACCGAAgcgACAGCCTACACGATTCCG	Mutation of Trp567 to Ala567
W567A-AS	GGCTGTcgcTTCGGTTGAAATGGCACG	

Small characters show amino acid mutations from tryptophan to alanine.

## Supplementary Materials

The following supporting information can be downloaded at: https://www.mdpi.com/article/10.3390/md20040250/s1, Table S1: BLAST search result for Uk-C; Figure S1: AA alignment of Uk-C and CBM79; Figure S2: AA alignment of Figure 8.
