# Supplementary material for: Characterization of an Unknown Region Linked to the Glycoside Hydrolase Family 17 β-1,3-Glucanase of Vibrio vulnificus Reveals a Novel Glucan-Binding Domain
Source: Mar Drugs. 2022 Mar 31;20(4):250. doi: 10.3390/md20040250 (PMC9026390; doi:10.3390/md20040250)
Supplement: Supplementary file 1 [file marinedrugs-20-00250-s001.zip › marinedrugs-1633370 -SI.pdf]

Figure S1. AA alignment of Uk-C and CBM79.

```
VvGH17_451-615                   SDADQFIVLDSALLTGGEAIAWEGTAYLAAEAGVLTLTTPPTAGTAKDWG
CBM79_WP_009984389.1_495-622     ---DGYTIKPNKKVTYSALGEDERMIGFSYKDFGISSSEKITEVQVNISA
CBM79_WP_009984389.1_663-790     ---DGFSIKPNQKVTYSALGEDERMIGFAYKDFGISSSDKITEVQVNISA
                                    *          *       *                  *

VvGH17_451-615                   WGAGVVLAGQAGANLTGFENGSLAFEIKGTTGSVLNIGFQTGLWGNNDRP
CBM79_WP_009984389.1_495-622     NKNIGKYVGQFGTSTTDSANGYWAMGDEITQSISGNSGTIT--WK---VP
CBM79_WP_009984389.1_663-790     NKNIGKYVGQFGTSTTDSANGYWAMGDEITQSISGNSGTIT--WK---VP
                                         ** *   *   **  *     *     * *  *  *     *

VvGH17_451-615                   QTNNFVVFGPTGRAISTEWTAYTIPMSELIKGNPDFSDVTSLIYFSGTAD
CBM79_WP_009984389.1_495-622     SDISSIIQTQYG--GEIKFGVWWIDCDEFT--------------------
CBM79_WP_009984389.1_663-790     SDISSIIQTQYG--GEIKFGVWWIDCDEFT--------------------
                                            *           *   *

VvGH17_451-615                   IDGGVVEVRNVVFNK
CBM79_WP_009984389.1_495-622     IDSVVLKT-------
CBM79_WP_009984389.1_663-790     IDSVVLKT-------
                                 **  *
```

Figure S2. AA alignment of Figure 8.

```
VvGH17_451-615                    ---------SDADQFIVLDSALLTGGEAIAWEGTAYLAAEAGVLTLTTPP
CBM79_WP_009984389.1_495-622      ------------DGYTIKPNKKVTYSALGEDERMIGFSYKDFGISSSEKI
CBM79_WP_009984389.1_663-790      ------------DGFSIKPNQKVTYSALGEDERMIGFAYKDFGISSSDKI
CBM72_ACA61140.1_349-547          --------AYHGSAFEGKYPVHDSSKGTVAFEGEKTLEWGEGVFVPSSML
CBM65_2YPJ                        ------------SGLVPRGSHMSSGADSGEIILFSGSNHADFKAWGGDDW
CBM4_1GUI                         ------------SINNGTFDEPIVNDQANNPDEWFIWQAGDYGISGARVS
CBM6_5FUI                         ----------SGNTLKIEAESYLYSNDVQKEPCSEGGENVGYINNGSWMS
CBM6_1W9T                         GSHMASDLKNPYERIQAEAYDAMSGIQTEGTDDDGGGDNIGWINDGDWVK
CBM22_1DYO                        ------KPEEPDAGYYYHDTFEGSVGQWTARGPAEVLLSGRTAYKGSESL
CBM85_MH727997_18-180             ----AEPQVPAGGQVLVAGEPSPDWTGRYEQGYSEVAQVQGPGFSRAVRV
CBM76_CAC83072.1_530-701          ----------QADAFDGLKTYEETKTETKTVEFTDIESMTNDKGFVVVPI
CBM78_4V17                        ----------------MGLAPLADGEKLYGKKGSEGTVTFTKAIGDNAFV

VvGH17_451-615                    TAGTAKDWGWGAGVVLAGQAGANLTGFENGSLAFEIKGTTGSVLNIG---
CBM79_WP_009984389.1_495-622      TEVQVNISANKNIGKYVGQFGTSTTDSANGYWAMGDEITQSISGNSG---
CBM79_WP_009984389.1_663-790      TEVQVNISANKNIGKYVGQFGTSTTDSANGYWAMGDEITQSISGNSG---
CBM72_ACA61140.1_349-547          TDVGEDVEVELTYKLDFTDYDDIQFMYNNGGWQKIPSGLSMDGKAFDGAD
CBM65_2YPJ                        PSAFEISPKYEPMKLDLNKNFEIKVDYNGADIVLIFARWDKDIWAQIS--
CBM4_1GUI                         DYGVRDGYAYIITIADPGTDTWHIQFNQWIGLYRGKTYTISFKAKADTPRP
CBM6_5FUI                         YPGINFPSSGNYLIEYRVASAVDGGRFSSDLEAGETVLGELSVPNTGG--
CBM6_1W9T                         YERVHFERDASSIEVRVASDTPGGRIEIRTGSPTGTLLGDVQVPNTGG--
CBM22_1DYO                        LVRNRTAAWNGAQRALNPRTFVPGNTYCFSVVASFIEGASSTTFCMKLQY
CBM85_MH727997_18-180             TTAERTAPWNVQFRVPVNAPVAKGDVLLLEFWARCLRTEDESGQGYIRAC
CBM76_CAC83072.1_530-701          EDAEHLSKIIINGAVTSSAGSSWATAGCAVCMNVTAKDGSGFWTYKSYNL
CBM78_4V17                        EIKTGADTGFMNGCLGFSESIDGKNYWVAYVWQTKKSDTISIDMSSPVQI

VvGH17_451-615                    -FQTGLWGNNDRPQTNNFVVFGPTGRAISTEWTAYTIPMSELIKGNPDFS
CBM79_WP_009984389.1_495-622      -TIT--WK---VPSDISSIIQTQYG--GEIKFGVWWIDCDEFT-------
CBM79_WP_009984389.1_663-790      -TIT--WK---VPSDISSIIQTQYG--GEIKFGVWWIDCDEFT-------
CBM72_ACA61140.1_349-547          FSASSVYGIQSGDTKTSVLTFDASAYGYVSKYEMVIQGHGVIMKKVTVRA
CBM65_2YPJ                        -PYYVVDGTAVFTKEQIAKAYGSDDFSGLDYIAVKPLPSEEGVT------
CBM4_1GUI                         INVKILQNHDPWTNYFAQTVNLTADWQTFTFTYTHPDDADEVVQISFELG
CBM6_5FUI                         ------WQNWTTVSQTVNVSAGTYQFGLYSISGGWNINWIRITK------
CBM6_1W9T                         ------WQQWQTVTGNVQIQPGTYDVYLVFKGSPEYDLMNVNWFVFRANG
CBM22_1DYO                        VDGSGTQRYDTIDMKTVGPNQWVHLYNPQYRIPSDATDMYVYVETADDTI
CBM85_MH727997_18-180             VEKRDRPWTKSLERTVTVGYRWQRFLLPCTCREDYGPGGLYLAFGTGQVP
CBM76_CAC83072.1_530-701          PLGSKSSATVKFDGIFTKETGEGADKVKEDLEATVAEGKVELQKWWDASE
CBM78_4V17                        AEIIGTETQEVTDADTIKKLTDKIKTEKSALLQVWYASDKTGKQIDPADS

VvGH17_451-615                    DV-TSLIYFSGTADIDGGVVEVRNVVFNK-----------------
CBM79_WP_009984389.1_495-622      --------------IDSVVLKT------------------------
CBM79_WP_009984389.1_663-790      --------------IDSVVLKT------------------------
CBM72_ACA61140.1_349-547          PSGTSAIPLIKADYPDNTIIYNLNGQRVETPRKGIYIQNGKKYIAQ
CBM65_2YPJ                        -------------VTKVSGIYTNGGSED------------------
CBM4_1GUI                         -------------EGTATTIYFDDVTVSPQ----------------
CBM6_5FUI                         ----------------------------------------------
CBM6_1W9T                         ----------------------------------------------
CBM22_1DYO                        N-----------FYIDEAIGAVAGTVI-------------------
CBM85_MH727997_18-180             ------------QVIEIGGIRLISYDKR------------------
CBM76_CAC83072.1_530-701          KSEESSEDKIEVKYSSIQVVYEYAQGEAPKV---------------
CBM78_4V17                        -------------ASESIEVYIPSASADEALE--------------
```
